# All Health Partnerships, Great and Small: Comparing Mandated With Emergent Health Partnerships: Comment on "Evaluating Global Health Partnerships: A Case Study of a Gavi HPV Vaccine Application Process in Uganda"

**DOI:** 10.15171/ijhpm.2017.68

**Published:** 2017-06-11

**Authors:** Elaine Green, Dan Ritman, Graeme Chisholm

**Affiliations:** ^1^ Independent Health and HIV&AIDS Consultant, Hampton, UK.; ^2^ Tropical Health and Education Trust, London, UK.

**Keywords:** Health Partnership, Mandated Partnerships, Emergent Partnerships, Social Network Analysis, Principles of Partnership

## Abstract

The plurality of healthcare providers and funders in low- and middle-income countries (LMICs) has given rise to an era in which health partnerships are becoming the norm in international development. Whether mandated or emergent, three common drivers are essential for ensuring successful health partnerships: trust; a diverse and inclusive network; and a clear governance structure. Mandated and emergent health partnerships operate as very different models and at different scales. However, there is potential for sharing and learning between these types of partnerships. Emergent health partnerships, especially as they scale up, may learn from mandated partnerships about establishing clear governance mandates for larger and more complex partnerships. By combining social network analysis, which can detect key actors and stakeholders that could add value to existing emergent partnerships, with Brinkerhoff ’s comprehensive framework for partnership evaluation, we can identify a set of tools that could be used to evaluate the effectiveness and sustainability of emergent health partnerships.

The plurality of healthcare providers and funders in low- and middle-income countries (LMICs) has given rise to an era in which health partnerships are becoming the norm in international development.^1^ The advent of the Sustainable Development Goals^2^ has further increased the importance of establishing well-functioning health partnerships. Building the strong and sustainable health systems required to achieve universal health coverage (UHC) requires global multi-stakeholder partnerships^3^ such as the UHC 2030 International Health Partnership and more focused ‘twinning’ arrangements^4^ between technical and research partners in high-income countries and LMICs. A question that arises at this point, however, is what does an effective health partnership really look like and can social network analysis act as a useful tool for systematically evaluating health partnerships.

Kamya and colleagues’^5^ article presents an example of a clear and well-structured partnership between multiple stakeholders, with a very explicit aim. Other types of health partnerships, however, are not always so clearly structured. Experience from the Tropical Health and Education Trust (THET) demonstrates how less-structured health partnerships can still deliver concrete benefits for LMIC health systems. Whilst there are stark differences between these two approaches, by assessing their comparative strengths and weaknesses it is possible to draw out lessons that can inform the development of future health partnerships.

## Defining Partnership Approaches

Gavi’s partnership in Uganda, as presented in the Kamya et al^5^ article, takes the form of what can be described as a ‘mandated’ partnership.^6^ Such partnerships have very clear and explicit aims and outcomes and are carefully managed to ensure effective inclusion and representation of the wide range of stakeholders required to achieve a successful outcome.

Mandated partnerships, however, are just one type of partnership being used to deliver sustainable health impacts in LMICs. A second type of health partnership is one that can be described as ‘emergent.’^6^ Adopted by THET in its Health Partnership Scheme, emergent partnerships develop organically from personal relationships between health workers and act as a model for improving health and health services based on ideas of co-development between actors and institutions from different countries. The partnerships are long-term but not permanent and are based on ideas of reciprocal learning and mutual benefits. Defined as a relationship between a health institution in a high-income country and a counterpart health institution in an LMIC, they aim to strengthen health services by working with partners to design and implement projects based on needs identified by the LMIC partner.

Whether mandated or emergent, there are common drivers that are essential for ensuring the success of health partnerships. Three such drivers, as identified through Kamya and colleagues’ evaluation of Gavi’s efforts in Uganda, are trust, a diverse and inclusive network, and a clear governance mandate. THET’s Principles of Partnership^7^ identify other drivers including the need for partnerships to be strategic, harmonised and aligned, organised and accountable, respectful and reciprocal, and effective and sustainable.

Kamya et al use social network analysis^5^ to generate some valuable insights but the approach has limitations. Kamya et al^5^ refer to “looking inside the black box” of partnership but it is not clear they have successfully done more than put some labels on the black box, for example “trust.” THET’s experiential approach provides more qualitative details.

## Trust in Emergent vs. Mandated Partnerships

Emergent health partnerships often form organically, with a broadly defined focus and a sense of potential. This contrasts to a mandated partnership which, as the Gavi example demonstrates, is established with a clear aim and expected outputs. Crucial to both partnerships, however, is the need for the partnership to be built on trust. In the emergent partnerships supported by THET this trust develops over time, with partners working together for 5, 10 or more years, during which they jointly respond to opportunities and health system needs as resources and circumstances allow. This facilitates the establishment of long-term, sustainable yet flexible partnerships that are responsive to local health system challenges.

A particular challenge of emergent health partnerships is the imbalances of power that can arise when one partner, usually the high-income country partner, has greater ownership of funding contracts, greater availability of resources, greater technical expertise, and has failed to address prevailing attitudes and expectations of partner institutions.^8^ This contributes, in turn, to a lack of trust and local ownership, thereby undermining the long-term sustainability of the partnership. It is essential, therefore, that in both emergent and mandated partnerships attention is paid to partnership development, the risks associated with the partnership, and the establishment of countervailing systems to mitigate such risks.

THET encourages emergent health partnerships to assess the strength of their partnership in a number of areas related to the Principles of Partnership, including the quality of communication, alignment of work with national and institutional plans, and a commitment to learning. Health partnerships then agree specific objectives to address the weaker areas. As Popp et al note “while networks as structures can be mandated, successful relationships cannot simply be mandated… a critical issue for practitioners to understand in regard to the longer-term effec­tiveness of a network, whether emergent or mandated, formal or informal, appears to be allowing time for trust and commitment to be built.”^6^ THET suggests “making” as well as “allowing” time. Mandated partnerships like the Gavi partnership may benefit from paying explicit attention to partnership development such as by maintaining relationships when there are no specific activities to undertake.

## Stakeholders in Emergent vs. Mandated Partnerships

Mandated partnerships such as that implemented by Gavi in Uganda are, by their nature, required to form diverse and inclusive networks to be successful. Consequently, mandated partnerships are able to survey the political landscape and ensure that all relevant actors are included within the partnership. Even within mandated partnerships, however, it can be challenging to effectively engage influential partners (such as the Ministry of Education and Ministry of Finance) as is evidenced in the Kamya et al^5^ study.

Emergent partnerships often develop from personal relationships thus rarely consider the institutional context. This risks overlooking important stakeholders, potentially undermining important contributors to effectiveness or sustainability, or duplicating other work.

Furthermore, the primary aim of the emergent health partnerships supported by THET is to strengthen the health workforce, in order to strengthen health services and outcomes. However, health system strengthening requires simultaneous attention to several health system elements, and there can be numerous constraints and confounding factors. So as they scale up or look for greater effectiveness, emergent health partnerships may be able to learn from the more deliberate approach to network creation taken by mandated partnerships.

The strategically-selected, diverse and inclusive membership of a mandated partnership (“Who do we need to make this happen?”) is something that emergent health partnerships can learn from. Perhaps emergent health partnerships can undertake stakeholder analysis (including social network analysis) to identify the agencies that can support the change they want to make, and then deliberately develop effective working relationships. This will be particularly important as emergent health partnerships look to scale up the innovative approaches they have developed.

## Governance Mandates in Emergent vs. Mandated Partnerships

Emergent partnerships typically have fewer partners than mandated partnerships. As a result they have simple structures, which makes decision-making easier and requires leaner governance mandates. Although sophisticated governance mechanisms are not a prerequisite, THET’s experience has demonstrated that even in emergent health partnerships it is critical to foster a strong sense of shared ownership. Achieving this requires transparency from the outset, and throughout the partnership. Particularly important is transparency of roles and responsibilities of all partners and budgetary transparency. With this transparency in place and being maintained, ownership among partners can be further reinforced by ensuring the inclusion of partner organisations and partner institutions at all stages of proposal design and programme implementation, promoting equitably shared responsibility, and fostering a strong sense of ownership through joint planning and implementation.^9^

Emergent health partnerships, especially as they scale up, may learn from mandated partnerships about establishing clear governance mandates for larger and more complex partnerships. Clear and transparent governance is particularly important for securing the necessary ownership from all relevant government, non-government and technical partners to ensure sustainability of the programme and for reinforcing alignment with local and national health priorities.

## Analytical Methods for Evaluating Health Partnerships

There is a dearth of rigorous evidence on the effectiveness of emergent health partnerships, “not surprising given institutional health partnerships do not lend themselves easily to case control studies and randomised control trials due to their high level of diversity and operation in complex social systems. There [is], however, a body of practice based on knowledge and experience.”^10^

The social network analysis used by Kamya et al^5^ offers a useful tool for evaluating the perceptions of the added value of health partnerships. By combining this with Brinkerhoff’s comprehensive framework^11^ for partnership evaluation we can identify a set of tools that could also be used to evaluate the effectiveness and sustainability of emergent health partnerships.

The Brinkerhoff framework^11^ suggests five dimensions to evaluate (context and partnership prerequisites; partnership structure; partnership process; partnership practice; and impact or added value) and proposes causal relationships between these. Through its experience of implementing nearly 200 successful emergent health partnerships, THET has identified eight essential drivers of effective partnerships. Whilst these principles of partnership have been developed, primarily, to guide good practice in the establishment and implementation of emergent health partnerships, they can be equally applicable to mandated partnerships. Table compares the two sets of concepts.

**Table:** Mapping the THET and Brinkerhoff Approaches to Partnership

**THET’s Principles of Partnership**	**Brinkerhoff Framework**
• Strategic • Harmonised and aligned	Context and partnership prerequisites
• Organised and accountable	Partnership structure
• Flexible, resourceful and innovative	Partnership process
• Respectful and reciprocal • Responsible • Committed to joint learning	Partnership practice
• Effective and sustainable	Impact/added value

Abbreviation: THET, Tropical Health and Education Trust.

## Conclusion

Mandated and emergent health partnerships operate as very different models and at different scales. However, there is potential for sharing and learning between these types of partnerships and there may be significant value in exploring this further. Trust, for example, is a critical factor for the success of both mandated and emergent partnerships, while both mandated and emergent partnerships experience challenges engaging influential stakeholders. Sharing lessons and approaches for addressing these challenges may help improve the effectiveness and sustainability of the two types of partnership. Mandated partnerships have clearer governance structures than emergent partnerships and, as emergent partnerships look to scale-up, they may find it useful to draw out lessons on governance and shared ownership from mandated partnerships.

## Acknowledgements

We would like to acknowledge the input of THET’s community of partnerships for their part in the development of Principles of Partnership and also Laura Macpherson for proof reading the manuscript.

## Ethical issues

Not applicable.

## Competing interests

Authors declare that they have no competing interests.

## Authors’ contributions

DR conceived this commentary and participated in the drafting of the manuscript. EG critiqued the conception and participated in the drafting of the manuscript. GC critiqued the conception and conceived the Principles of Partnership. All authors read and approved the final manuscript.

## Authors’ affiliations

^1^Independent Health and HIV&AIDS Consultant, Hampton, UK. ^2^Tropical Health and Education Trust, London, UK.
